# Rapid Identification of Major QTLs Associated with Rice Grain Weight and Their Utilization

**DOI:** 10.1371/journal.pone.0122206

**Published:** 2015-03-27

**Authors:** Feifei Xu, Xiao Sun, Yaling Chen, Yan Huang, Chuan Tong, Jinsong Bao

**Affiliations:** Institute of Nuclear Agricultural Sciences, College of Agriculture and Biotechnology, Zhejiang University, Huajiachi Campus, Hangzhou, 310029, P. R. China; Mahatma Phule Agricultural University, INDIA

## Abstract

To uncover the genetics of rice grain weight, we constructed an RIL population derived from a cross between a large grain accession M201 and a small size variety JY293. Specific Locus Amplified Fragment Sequencing (SLAF-Seq) technology was used to genotype two bulked DNA pools made from individual DNA of the heaviest 30 lines and the lightest 30 lines according to the 1000 grain weight (TGW). Bulked segregant analysis (BSA) was used to identify SLAFs strongly associated with TGW. Two marker-intensive regions at 24,600,000–24,850,000 bp and 25,000,000–25,350,000 bp on chromosome 3 were identified tightly related to the TGW. Then a linkage map of chromosome 3 was constructed with SSR markers and some SLAF derived single nucleotide polymorphisms (SNPs). Quantitative trait locus (QTL) mapping for TGW, grain length, grain width, and grain thickness revealed one major QTL in the second hot-region and two other minor QTLs for grain weight. These three QTLs displayed hierarchical effects on grain length and grain weight in order of *qTGW3*.*2* (*qGL3*) *qTGW3*.*1* (*GS3*) *qTGW3*.*3*. Multiple comparisons of means among the eight combinations of 3 QTLs revealed that the lines with two of three QTLs deriving from M201 displayed a large grain weight phenotype (TGW 40.2g, average data of three years) and lines with both *qTGW3*.*1* and *qTGW3*.*3* alleles from M201 (42.5g) had similar grain weight to the *qTGW3*.*2* (40.8g) alone. Two strategies with similar effectiveness were proposed to improve grain weight by marker-assisted selection (MAS). One is to introduce the novel *qTGW3*.*2* allele alone, and the other is to pyramid *qTGW3*.*1* and *qTGW3*.*3* alleles together. One new allele of *GS3* (39 bp deletion in intron 1) and two SNPs in coding sequence of *qGL3* identified in this study from M201 are useful in pyramiding elite alleles for molecular breeding for improvement of rice yield.

## Introduction

Rice (*Oryza Sativa*. L) is one of the most important cereals and feeds more than half of the world’s population. Breeding high yield rice is always the target of plant breeders. The introduction of semi-dwarf rice (*Oryza sativa* L.) and hybrid rice breeding has led to yield increase throughout Asia since the 1960s. However, there is a bottleneck in further increasing grain yield by traditional hybridization breeding method due to limited knowledge on genetics of the grain yield traits. Exploiting new QTLs for grain yield is always a hot topic for improving rice grain yield with marker assisted selection (MAS).

Grain weight is the direct trait for rice grain yield, and is controlled by cell division in outer glumes and grain filling rate [1]. *GS3* and *qGL3* negatively regulate cell division in outer glumes, so that the loss of their functions increase grain yield [2–5]. In sink organs, the *tgw6* allele affects the timing of the transition from the syncytial to the cellular phase by controlling IAA supply and limiting cell number and grain length [6]. *GW2*, *GW5* (*qSW5*) and *GS6* negatively regulate grain width, and loss of their functions lead to the increased grain width [7–10]. Conversely, *GS5* and *GW8* are positive regulators. Mutations in the promoter sequence of *GS5* and *OsSPL16* lead to increased gene expression and hence increased grain width [11,12]. *GIF1* encodes the cell-wall invertase required for carbon partitioning during early grain-filling [13]. Another QTL for grain filling, *FLO2*, is a pleiotropic gene that plays a pivotal regulatory role in rice grain size and starch quality by affecting storage substance accumulation in the endosperm [14]. All of these genes were isolated by biparental cross linkage mapping methods. However, it is labor intensive and time-consuming to construct linkage maps to finely map the putative gene.

The draft genomic sequences of two rice subspecies, *O*. *sativa ssp*. *japonica* (cv. Nipponbare) and *O*. *sativa ssp*. *indica* 93–11, have provided us with a vast amount of information on the rice genome and allowed us to perform detailed genetic analysis [15–17]. Next- generation sequencing (NGS) technologies have emerged as an important tool for genetic analysis, providing unprecedented wealth of high resolution genotype information that enables many traditionally difficult, time-consuming and expensive genetic assays to be supplanted by rapid and relatively cheap assays [18]. NGS has been widely used in polymorphism discovery, mutation mapping, transcriptome sequencing, analysis of DNA-protein interactions through Chip-seq, and genome-wide detection of DNA methylation [19]. In combination with bulked segregant analysis (BSA), NGS derived methods, such as X-QTL, MutMap, QTL-Seq, SHOREmap and NGM, have accelerated the speed in detecting QTLs for complex traits or physical locations for mutations [18,20–23].

Specific-locus amplified fragment sequencing (SLAF-seq) is an efficient method of large-scale genotyping, which combines locus-specific amplification and high-throughput sequencing to reduce the complexity of the genome [24]. SLAF-Seq has emerged as an effective method in developing specific molecular markers. It allows researchers to design the experimental system through bioinformatics and screen for fragments of a specific length from the constructed SLAF-seq libraries [25]. The application of SLAF-seq technology has been successful in developing specific molecular markers for *Thinopyrum Elongatum* and common carp with high quality SLAFs [24,25].

In this study, SLAF-Seq technology was first used to sequence the large grain and small grain bulked segregate DNA samples and the two parental DNA samples. Then, linkage map was constructed for QTL mapping based on results from SLAF-seq and the markers derived from the hot-region SLAFs. There are two main objectives in this study, one is to quickly identify QTLs related to grain weight by a combination of SLAF-Seq with linkage mapping, and the other is to propose two practical protocols with similar effectiveness to improve grain weight by MAS.

## Materials and Methods

### Construction of a RIL population and traits measurement

The parental line, Jiayu 293 (JY293) is a small grain *indica* variety, and the other parental line, M201 is an extremely large grain *indica* variety. The recombined inbred lines (RILs) were derived from a cross M201 × JY293. Two hundred and thirty four lines along with both parents were planted from May to October in 2009, 2012 and 2013 at 7^th^, 8^th^ and 9^th^ generation at Zhejiang University farm, Hangzhou, China.

After being air-dried and stored at room temperature for 3 months, three hundred fully filled grains were selected from each RIL and their parents for the measurements of grain traits. 1000-grain weight (TGW) was evaluated from 100—grain weight, and grain length (GL), grain width (GW) and grain thickness (GTH) were measured with a vernier caliper. Grain length to width ratio (GLW) was a derived trait (GLW = GL/GW). The mean values of 100- grain weight for three replications and the mean values of GL, GW, GTH and GLW for ten replicates were used as input data to identify QTLs (S1 Table).

### DNA preparation for SLAF-Seq

**Fig 1:**
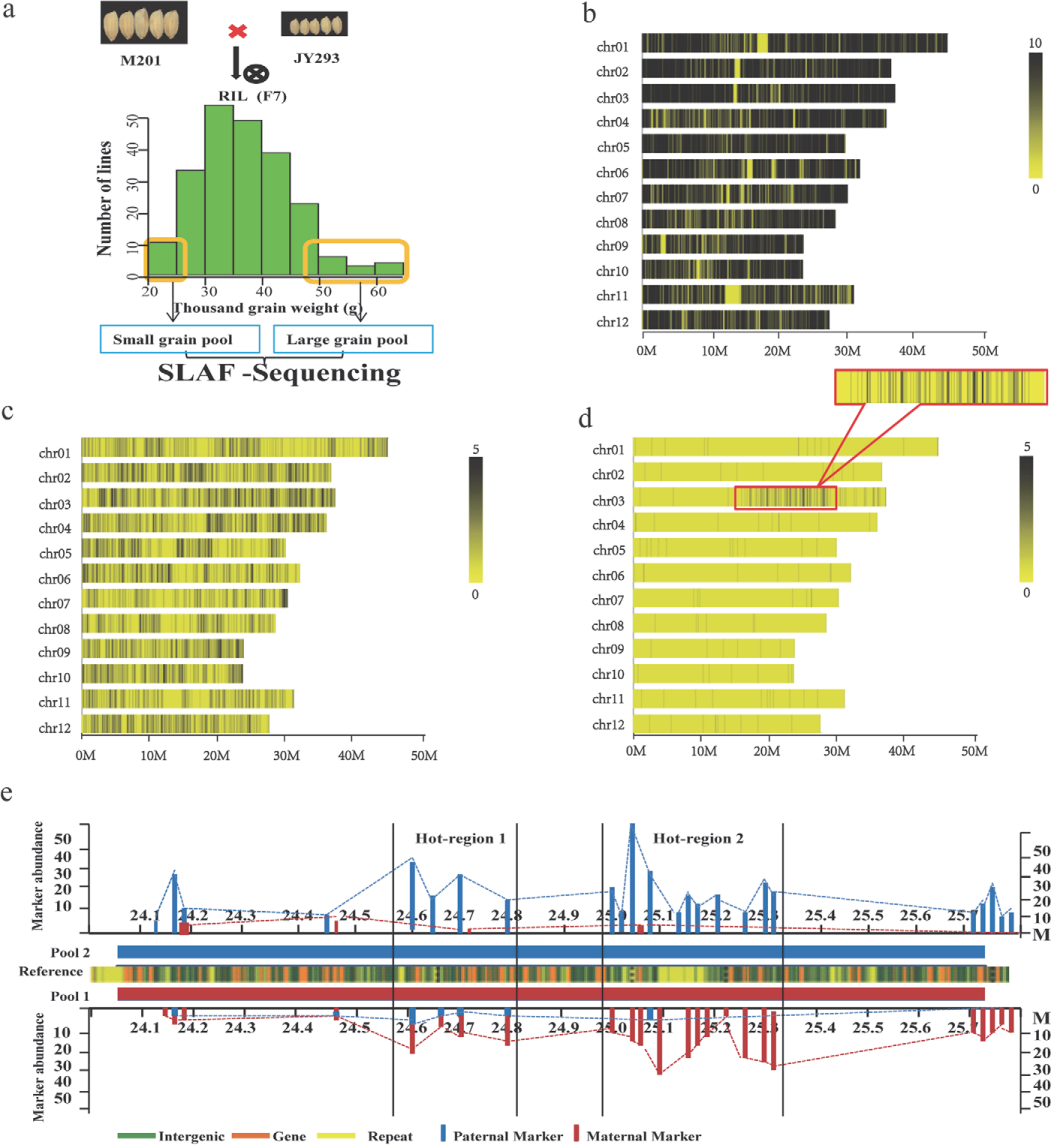
A simplified scheme for SLAF-Seq. (a) Thirty lines with largest TGW and 30 lines with lightest TGW were pooled and used for SLAF-Sequencing (the histogram was drawn based on the data collected at the F_7_ generation) (b) All SLAFs (black lines) distributed on 12 chromosomes (c) Polymorphic SLAFs (black lines) distributed on 12 chromosomes (d) Polymorphic markers (black lines) between two DNA bulks distributed on 12 chromosomes with intensive markers distributed in the block of chromosome 3. (e) Identification of the hot region for TGW. The reference genome is in the middle of the map, the green box means intergenic regions, orange box means gene regions, yellow box means repeat regions. Red and blue lines represent the relative abundance of the maternal and paternal alleles between two bulked DNA pools (Pool 1 and Pool 2).

According to the TGW, the heaviest 30 lines were selected as the large grain group, and the lightest 30 lines were selected as the small group from the 234 F_7_ RILs (2009, Hangzhou, China) (Fig. 1A, S1 Table). The DNA from each line of each group was extracted following the CTAB procedure [26] and was purified by RNase. DNA quality and concentration were measured by 1.0% agarose gel electrophoresis, and adjustments were made for a final DNA concentration of 100 ng μL^-1^ and the total DNA over 20 μg. Two DNA bulks were established by equally mixing the 30 individual genomic DNA from each group, respectively.

### SLAF library construction and sequencing

The construction of SLAF library followed the methods proposed by Sun et al [24] with little modification. In short, genomic DNA of two DNA bulks and two parents were incubated at 37°C with *Mse*I (New England Biolabs, NEB), T4 DNA ligase (NEB), ATP (NEB), and *Mse*I adapter. Restriction-ligation reactions were heat-inactivated at 65°C for 10 min, and then digested for additional restriction enzyme *Hae*III at 37°C. The PCR reaction was performed using diluted restriction ligation samples, dNTP, Taq DNA polymerase (NEB) and *Mse*I primer containing barcode 1. The PCR products were purified using EZNAH Cycle Pure Kit (Omega) and pooled. The pooled sample was incubated at 37°C with *Mse*I, T4 DNA ligase, ATP and Solexa adapter. The sample was purified using a Quick Spin column (Qiagen), then run out on a 2% agarose gel. Fragments with 380–450 bp in size were isolated using a Gel Extraction Kit (Qiagen). These fragments were used in PCR amplification with Phusion Master Mix (NEB) and Solexa amplification primer mix. Phusion PCR settings followed the Illumina sample preparation guide. Samples were gel-purified, and products with appropriate sizes (380–450 bp) were excised and diluted for sequencing by Illumina GAIIx (Illumina, San Diego, CA, USA).

### SLAF sequence comparison, polymorphic analysis and identification of the associated markers

The massive sequences were then obtained and analyzed using SLAF_Poly.pl (Biomarker, Beijing, China). After a sequence comparison using BLAT [27], sequences with over 90% identity were grouped in one SLAF locus and a large number of specific fragments were selected for specific molecular markers development.

These selected reads with entire depth >10 and depth >5 (only those similar to the depth over 10) were compared to the reference genome [28]. Polymorphic SLAFs and markers were summarized and compared among the two parents and the two bulked DNA samples in Table 1. Polymorphic SLAFs refer to SLAFs that show polymorphic between the two parents, and markers refer to these SLAFs show polymorphic between the two bulked DNA samples. The relative marker abundance in bulked DNA pool 1 (the heaviest pool) was calculated as the number of reads of maternal allele divided by the number of reads of paternal allele, whereas in pool 2 (the lightest), the relative marker abundance was calculated as the number of reads of paternal allele divided by those of maternal allele. If one allele had zero read, the relative abundance was set at 50. It is expected that the larger the relative abundance was, the higher possibility the marker was associated with TGW.

**Table 1:** SLAFs data summary for parents and bulked DNA pools.

**Total reads**	
No. of reads	6,985,489
Reads in depth> = 5	62,903
Reads in depth 10~1000	53,409
Average depth of reads depth range 10~1000	49.33
**High quality SLAFs**	
No. of SLAFs	40,114
SNP	3,525
EnzymePos SNP (EP-SNP)	132
InDel	285
No Polymorphism	35,433
Unknown	650
Repeat	89
**Polymorphic SLAF Number**	3,942
**Polymorphic markers between two bulked DNA pools**	
No of markers	227
SNP Marker	202
InDel Marker	16
EP-SNP Marker	9

SNP: single nucleotide polymorphism. EnzymePos SNP: SNP at restriction enzyme cleavage position. InDel: insertion/deletion

### Development of CAPS, dCAPS, and InDel markers and genotyping of the RILs

In order to minimize the genetic interval for fine mapping and to verify the accuracy of SLAF-Seq, the development of the cleaved amplified polymorphic sequences (CAPS) and derived CAPS (dCAPS) for the SNPs generated from SLAF-seq and functional sites of *qGL3* followed the methods of [29,30] and two InDel (Insert-Deletion) markers for *GS3* and SLAF13474 were developed for genotyping. The PCR was carried out in a total volume of 20 μL containing 10 mM Tris-HCl (pH 9.0), 50 mM KCl, 0.1% Triton X 100, 2 mM MgCl_2_, 0.1 mM dNTPs, 200 nM primers, 1 unit of *Taq* polymerase, and 30 ng of genomic DNA. All amplifications were performed on a PTC-100 thermal cycler (MJ Research, Inc.) under the following conditions: 5 min at 95°C; 30 cycles of 30 s at 95°C, 30 s at 55°C and 40 s at 72°C; and a final extension step at 72°C for 10 min. Amplified PCR products were digested using suitable restriction endonucleases in a total volume of 20 μL according to the manufacturer’s instructions. The digests were resolved by electrophoresis in 1.5–2.0% agarose gel or PAGE gel and visualized on a Versa Doc (Bio-Rad) after staining with ethidium bromide.

### Linkage map construction and QTL mapping

A total of 100 simple sequence repeat markers (SSRs) evenly distributed on chromosome 3 were first used to select polymorphic markers among the two parents and two bulked DNAs. Finally, five markers developed from SLAFs, two functional markers derived from *GS3* and *qGL3*, and 25 SSRs showed polymorphism between the two parents were used to detect the genotype of all the RILs. Construction of linkage map and subsequent QTL detection by interval mapping were carried out by QTL IciMapping v 3.3.

### Statistic analysis

All the statistic analyses were carried out with the SAS program 9.1 (SAS Institute Inc, Cary Nc). Pro Corr was used to examine correlations between these traits. Analysis of variance (ANOVA) was carried out to using the general linear model procedure (Proc glm) and Duncan’s new multiple-range test was used to determine significant differences.

## Results and Discussion

### SLAFs polymorphism analysis and rapid identification of hot-spot regions

A total of 6,985,489 fragments were selected to obtain high quality SLAFs after two rounds of sequencing and exclusion of the low-quality fragments (Table 1). Tags with depth over five were aligned with the reference genome, and 40, 114 SLAFs were finally selected for further analysis (Fig. 1B). Totally, 3,942 SLAFs showed polymorphism between the two parents (Fig. 1C). A total of 227 polymorphic markers, including 202 SNP markers, 16 InDel markers and 9 EPSNP markers showed polymorphism between the two bulked DNA pools (Fig. 1D). Among these, 135 (59.47%) were located on chromosome 3, and 115 markers of which were located in the region 15~30M (Fig. 1D). It was found that there were two candidate hot-spot regions, 24,600,000~24,850,000 bp and 25,000,000 ~25,350,000 bp, which were highly associated with grain weight (Fig. 1E). It seemed that the second hot-spot region showed a stronger association with TGW, because the number of marker and their relative abundance in hot-spot region 2 were higher than those in hot-spot region 1 (S2 Table, Fig. 1E).

SLAF-seq is a recently developed high-resolution strategy for large scale de novo discovery and genotyping of SNPs [31]. Sun et al.[24] performed a pilot study on rice and soybeans, and selected 21,000 and 76,000 SLAFs by *Hae*III and *Mse*I digestion and purification restriction fragments. Compared to our study, we got 40, 114 SLAFs excluding low quality SLAFs, suggesting a high resolution sequence data was obtained in our study. Recently, SLAF-Seq has been successfully used in the employment to obtain sufficient markers to construct high-density genetic maps for sesame and soybean [31,32].

In the present study, we first used SLAF-seq technology combined bulked segregant analysis (BSA) to detect polymorphic markers between the two bulked DNA pools, and quickly indentified two marker intensive hot-regions for grain weight on chromosome 3. This method promoted the speed in detecting major QTLs on the whole genome-wide level. These polymorphic SLAF markers are also of great importance to minimize genetic interval in fine mapping and gene cloning (S3 Table, S1 Fig.).

### Linkage map construction and QTL mapping

**Fig 2:**
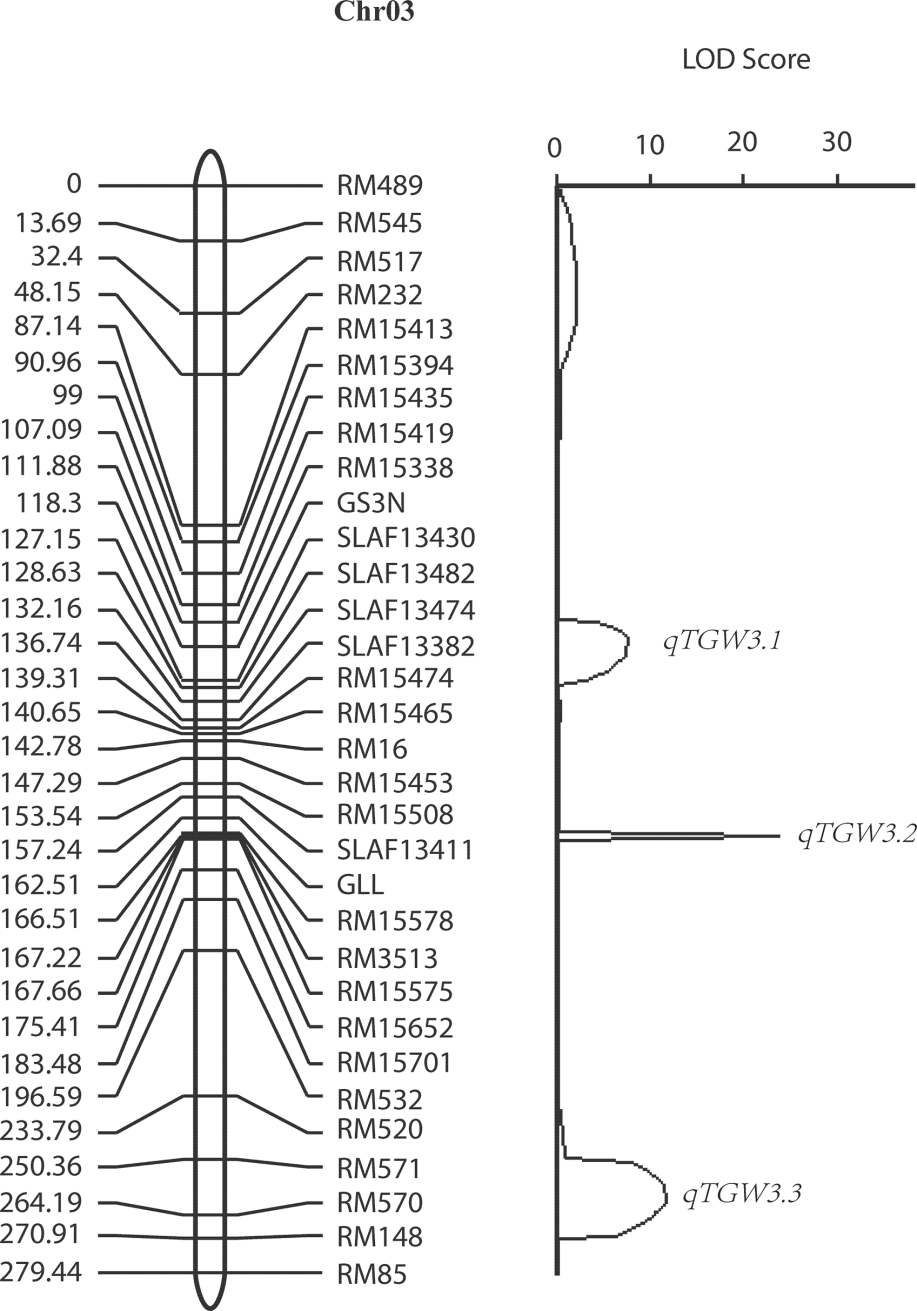
Genetic linkage map of chromosome 3 and QTL mapping for TGW. The linkage map was constructed with 32 markers, covering 179.4 cM, with an average distance of the adjacent markers of 5.61 cM (left); interval mapping for TGW was conducted and a plot of LOD scores was shown with three distinct peaks corresponding to *qTGW3*.*1*, *qTGW3*.*2* and *qTGW3*.*3* (right).

Due to two candidate gene hot-regions identified on chromosome 3, a linkage map of chromosome 3 using all 234 lines was constructed to detect and confirm major QTLs for grain weight. The linkage map was constructed with 32 markers, covering 179.4 cM, with average distance of the adjacent markers of 5.61 cM (Fig. 2).

Two QTLs for the same trait with one common marker was considered to be one QTL, we detected three QTLs for each trait of 1000-grain weight (TGW), three for grain length (GL) and three for grain length to width ratio (GLW) in 2009, 2012 and 2013. However, no QTL was detected for grain width (GW) on chromosome 3 with the three years data (Table 2), which might be controlled by QTLs other than chromosome 3, for example, *GW2* [7] and *GW5* [9]. The three QTLs were mainly located in three regions (I: 117-120cM, II: 168-169cM and III: 260-261cM) on the linkage map, in other words, these three QTLs played pleiotropic effects on the TGW, GL and GLW. (Fig. 2).

**Table 2:** QTLs for grain shape and grain weight detected on chromosome 3.

Trait	QTL	Interval	2009		2012		2013	
			LOD	PVE^a^(%)	LOD	PVE(%)	LOD	PVE(%)
**TGW**	*qTGW3*.*1*	RM15338~GS3PSTI	6.82	7.89	7.30	9.31	6.05	8.48
	*qTGW3*.*2*	RM15578~RM3513	19.05	21.80			8.58	12.19
		GLL~RM15578			15.14	19.96		
	*qTGW3*.*3*	RM571~RM570	9.27	11.88	5.60	7.86	3.83	6.74
**GL**	*qGL3*.*1*	GS3PSTI~SLAF13430	16.26	13.86				
		RM15338~GS3PSTI			18.78	17.60	5.94	9.97
	*qGL3*.*2*	RM15578~RM3513	36.02	33.59	27.78	26.51	10.46	16.17
	*qGL3*.*3*	RM571~RM570	11.27	9.70	11.65	11.33	3.33	5.60
**GLW**	*qGLW3*.*1*	GS3PSTI~SLAF13430	9.40	12.21	10.30	14.97		
	*qGLW3*.*2*	RM15575~RM15652	19.72	25.88				
		GLL~RM15578			10.44	14.45		
		RM15578~RM3513					4.92	10.39
	*qGLW3*.*3*	RM570~RM148	3.93	4.41	3.37	4.58		

^a^ PVE: Phenotypic variation explained by the QTL

*qTGW3*.*1*, *qGL3*.*1* and *qGLW3*.*1* which shared one molecular marker (GS3PSTI) were located in the region I. The molecular marker, GS3PSTI, is a CAPs marker that derived from the functional site of *GS3*, indicating that QTLs in this region is the *GS3*. *GS3* is a major QTL for grain length, and a minor QTL for grain width and thickness [2]. There is a C/A mutation in the second exon in which the A allele resulted in a premature stop, thus leading to the large grain phenotype. In addition to the functional marker GS3PSTI for *GS3*, we also detected a 39bp deletion in the first intron of the parental line M201, which is tightly linked to the functional site (S2 Fig.). As expected, the GS3N marker based on this InDel co-segregated well with the functional site and can be used in MAS.

*qTGW3*.*2* and *qGL3*.*2* flanked by RM15578 and RM3513, and *qGLW3*.*2* flanked by RM15575 and RM15652 were located in region II. *qTGW3*.*2* (*qGL3*.*2*, *qGLW3*.*2*) with the largest effect on the corresponding phenotype, was close to the second candidate hot-region (25,000,000 ~25,350,000 bp). Around this region, Zhang et al. [3] detected a major QTL for grain length that roughly flanked by the SSR marker RM15578. Since this marker was also in our linkage map, it was plausible that the QTL *qTGW3*.*2* identified in this study might be the *qGL3* gene. We sequenced the full-length of *qGL3* with 12 pairs of primers covering all exons and found that four SNPs (+1092, +1495, +2063, + 2838) in the exon regions of *qGL3* DNA sequence. Two SNPs at +1092 (C-A) and +1495 (T-C) resulted in amino acid substitutions, i.e. 364 aspartic acid-glutamic acid (364D-E), and 499 tyrosine-histidine (499Y-H). The other two did not cause amino acid changes (S4 Table). Compared to previous studies, all of the four large grain accessions shared the A allele at +1092 [3–5]. In this study, the M201 allele at +1495 had C allele. However, in other studies, the small grain accessions also had the +1495 C allele, indicating that this SNP at +1495 (T-C) with amino acid substitution had no effect on grain size. According to the functional site at +1092, we developed one dCAPs that could quickly identify *qGL3* functional site genotype with the restriction enzyme *Acc*I (S2 Fig.), which could be useful in MAS for selecting the A allele or eliminating the C allele to produce high yield varieties (S2 Table; S2 Fig.).

*qTGW3*.*3*, *qGL3*.*3* and *qGLW3*.*3* flanked by RM570 and RM571 were located in region III, which was correspondingly closed to the QTL for grain weight detected by Tang et al. [33].

### Correlation analysis and analysis of variance among grain weight related traits

Rice grain weight is mainly contributed by GL, GW and grain thickness (GTH). The grain weight related traits were stably inherited; the coefficient of correlation of the same trait between the two years was ranged from 0.608 for GW to 0.928 for GL (S5 Table). TGW was positively correlated with the other three traits in both years. GL was positively correlated with TGW and GLW in both years. Grain width was positively correlated with TGW, but negatively correlated with GLW.

Since grain thickness has large error rate during measurement, QTLs for GL and GW were easier to be identified than for GTH. ANOVA indicated that all of the three QTLs/genes significantly contributed to TGW, GL and GLW, but none was for GW (S6 Table). These three QTLs together could explain 43% for TGW, 60% for GL and 40% for GLW of total variation, respectively. Among the 3 QTLs, *qTGW3*.*2* had the largest contribution to TGW (36.7%), GL (45.3%) and GLW (40.7%). The *qTGW3*.*1* was followed, which could explain 31.4%, 32% and 36.7% for TGW, GL and GLW respectively. However, *qTGW3*.*3* had relatively less contribution to the variation of TGW (31.9%), GL (22.6%) and GLW (22.6%).

### Strategies in pyramiding elite alleles for MAS

A total of 201 RILs were classified into 8 groups based on the genotypes of GS3PST1, GLL and RM571 markers, representing the three main QTLs for grain weight and shape (Table 3). Groups 1, 2, 3 and 4 contained at least 2 of alleles deriving from the large grain parent M201, whereas Group 5, 6, 7 and 8 contained at least 2 of alleles coming from JY293.

**Table 3:** Multiple comparison of genotype class based on *qTGW3*.*1* (GS3PSTI), *qTGW3*.*2* (GLL) and *qTGW3*.*3* (RM571).

Group	*qTGW3*.*1* (C/A)^a^	*qTGW3*.*2* (C/A) ^a^	*qTGW3*.*3* ^*b*^		TGW^c^			GL^c^			GLW^c^	
				2009	2012	2013	2009	2012	2013	2009	2012	2013
1	A	A	1	46.44a	45.59a	40.18a	11.85a	12.19a	10.59a	3.57a	3.47a	3.12abc
2	A	A	2	41.99ab	41.89a	36.69ab	11.16ab	11.37b	10.16a	3.53a	3.39a	3.23ab
3	A	C	1	43.33a	43.34a	40.84a	10.32c	11.03b	10.40a	2.88bc	2.91c	3.04bc
4	C	A	1	42.33ab	43.04a	37.12a	10.77bc	10.75b	9.80ab	3.18b	3.10c	2.98bc
5	A	C	2	33.96cd	36.59b	31.73c	9.23d	9.52c	9.02bc	2.95bc	2.95c	2.89bc
6	C	A	2	37.69bc	35.10b	32.17bc	9.10de	9.12cd	9.92ab	2.62c	2.52e	3.54a
7	C	C	1	35.34cd	34.24b	30.91c	9.14de	9.33cd	9.06bc	2.89bc	2.89c	2.93bc
8	C	C	2	31.49d	31.84b	29.23c	8.38e	8.56d	8.42c	2.65c	2.62e	2.71c

^a^ C/A represents the functional SNP in the target gene of parent M201 (A) and JY293 (C).

^b^ 1 represents the allele comes from M201, 2 represents the allele comes from JY293

^c^Different letters indicate significant difference at *P*<0.05.

For TGW, Group 1, 2, 3 and 4 were significantly larger than those of Group 5 to 8. Group 6 with *qTGW3*.*2* allele from M201 had no significant difference with group 2 in 2009 and 2013. For GL, Group 1 had the largest GL, and was significantly different with group 3 and group 4 in 2009 and 2012. As expected, there was no difference among 8 groups for GW, as these three QTLs had no effect on grain width. For GLW, Group 1 and Group 2 had no significant difference with each other, but had significant difference with the other 6 groups. Compared to other 3 groups with at least two alleles from JY393, Group 6 with only *qTGW3*.*2* allele from M201, had the largest TGW. As expected, Group 8 with all alleles coming from JY293 produced smallest TGW, GL and GLW.

Multiple comparisons of the three QTLs among the RIL lines provide us a good example for pyramiding minor QTLs together to produce large grain rice. *qTGW3*.*2* had the largest contribution to TGW, followed by *qTGW3*.*1*, and then *qTGW3*.*3* (Table 2, S6 Table). Among the four large grain groups, three of them contained *qTGW3*.*2* allele from M201. The only exception, Group 3, which contains two minor alleles of *qTGW3*.*1* and *qTGW3*.*3* had the phenotype of large grain. The grain size of Group 3 was even larger than Group 6 which contains the major QTL (*qTGW3*.*2*) alone. Group 6 had no significant difference from Group 5 and Group7. The mutation of *qTGW3*.*2* (*qGL3*) at +1092 (C-A) is rarely existed and is scarcely utilized in modern rice breeding practice [3]. However, *GS3* has been widely existed in the cultivars and *qTGW3*.*3* is a new QTL that will be useful in breeding programs [34].

Therefore, along with the markers developed from mutation (C-A) at +1092 of *qTGW3*.*1*, we proposed two protocols for breeding large grain rice accessions. One is to introduce the *qTGW3*.*2* into other rice accessions by MAS to form the super-large grain varieties; the other is to produce large grain varieties by pyramiding *qTGW3*.*1* and *qTGW3*.*3* to produce very large grain varieties. It should be noted that others reports have not considered three QTLs together [3–5], and their applications have not yet indicated.

## Conclusions

The application of SLAF-seq in combination with the traditional linkage mapping and bulked segregant analysis have succeeded in detecting the major QTLs responsible for grain weight related traits. The procedures for detecting major QTL by SLAF-seq and linkage mapping were as follows: (I) Marker identification from polymorphic SLAFs between the two bulked DNA pools, (II) High density markers region and hot-region identification which may be strongly associated with the target trait, (III) Linkage map construction of the candidate chromosome, and (IV) QTL mapping to confirm the hot-region or to delimit it to a narrower region.

*qTGW3*.*1* (*GS3*), *qTGW3*.*2* (*qGL3*) and q*TGW3*.*3* displayed hierarchical effects on grain length and grain weight in order of *qTGW3*.*2*> *qTGW3*.*1> qTGW3*.*3*. Two ways with similar effectiveness are proposed to improve grain weight by MAS. The one is to use the novel *qTGW3*.*2* allele, and the other is to pyramid both *qTGW3*.*1* and *qTGW3*.*3* alleles. Since the strategies proposed for TGW improvement are based on analysis of a single mapping population, more validations with other breeding populations are needed. The new allele of *GS3* (39 bp deletion in intron 1) and two SNPs in coding sequence of *qGL3* identified in this study from M201 are useful in performing the validation and future molecular breeding for improvement of rice yield.

## Supporting Information

S1 Fig Gel image showing SLAF-SNPs among the recombined inbred lines. (a) SLAF13382 (b) SLAF13411 (c) Indel13474 (d) SLAF13482.
(TIF)

S2 Fig Gel images and primers for the development of the functional markers of rice grain shape. a: An InDel marker developed for 39bp deletion in *GS3* shown in red between the large grain (M201) and the small grain (JY293) rice. b: A dCAPs primer introduced 1 bp mutation (g in green color) resulting in a cleavage site of restriction enzyme *Acc*I for detection of the functional SNP of *qGL3*.
(TIF)

S1 Table Descriptive statistics of the rice grain weight related traits in parents and RIL population observed in 2009, 2012 and 2013 (SD standard deviation). (DOCX)

S2 Table No. of candidate genes in the hot regions. (DOCX)

S3 Table CAPS, dCAPs and InDel markers used for linkage mapping. (DOCX)

S4 Table SNPs detected in the coding sequence of *qGL3*. (DOCX)

S5 Table Correlation analysis among rice grain weight related traits (under the diagonal: 2009, upper the diagonal: 2012, the Coefficient of the same trait between the two years is shown in diagonal in bold). (DOCX)

S6 Table ANOVA analysis of the major QTLs for grain yield related traits (2009). (DOCX)
