# Supplementary figures and images for: Rapid Identification of Major QTLs Associated with Rice Grain Weight and Their Utilization
Source: PLoS One. 2015 Mar 27;10(3):e0122206. doi: 10.1371/journal.pone.0122206 (PMC4376791; doi:10.1371/journal.pone.0122206)

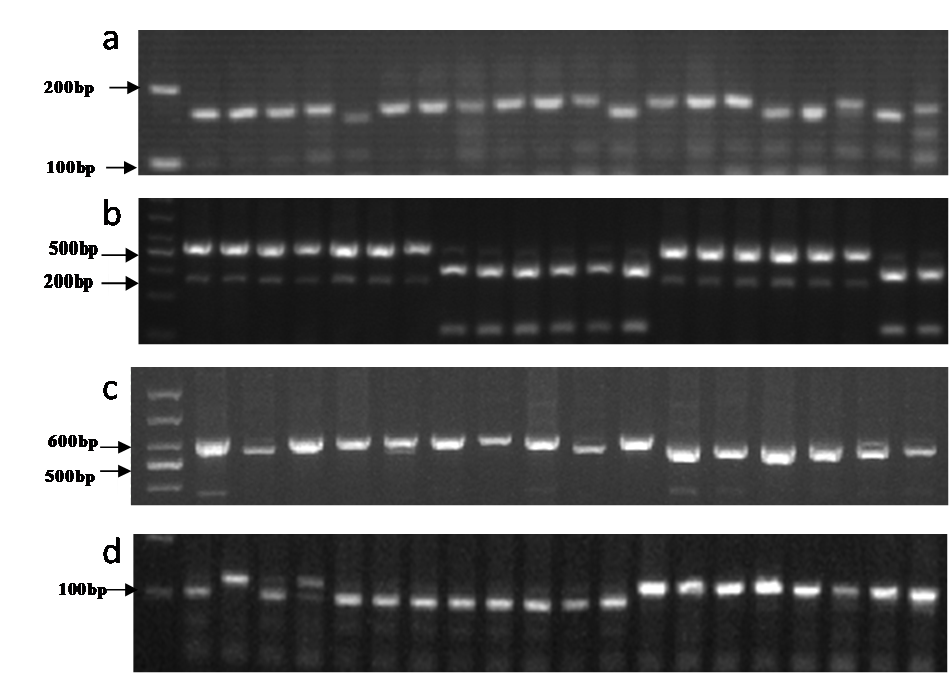

Supplement: S1 Fig — (a) SLAF13382 (b) SLAF13411 (c) Indel13474 (d) SLAF13482. (TIF) [file pone.0122206.s001.tif]

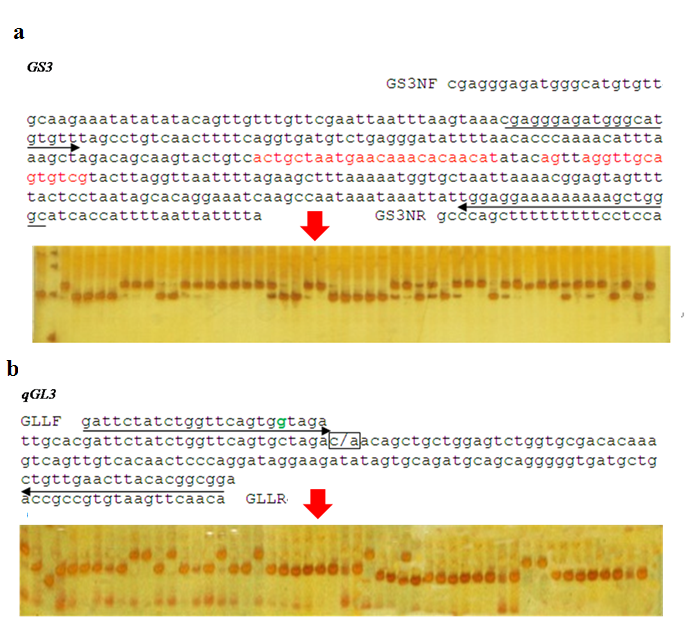

Supplement: S2 Fig — a: An InDel marker developed for 39bp deletion in GS3 shown in red between the large grain (M201) and the small grain (JY293) rice. b: A dCAPs primer introduced 1 bp mutation (g in green color) resulting in a cleavage site of restriction enzyme AccI for detection of the functional SNP of qGL3. (TIF) [file pone.0122206.s002.tif]
