# Supplementary material for: Rapid Identification of Major QTLs Associated with Rice Grain Weight and Their Utilization
Source: PLoS One. 2015 Mar 27;10(3):e0122206. doi: 10.1371/journal.pone.0122206 (PMC4376791; doi:10.1371/journal.pone.0122206)
Supplement: S1 Table — (DOCX) [file pone.0122206.s003.docx]

**S1 Table.** Descriptive statistics of the rice grain weight related traits in parents and RIL population observed in 2009, 2012 and 2013 (SD standard deviation)

| **Trait** | **Year** | **Parents** | |  | **RIL population** | |
| --- | --- | --- | --- | --- | --- | --- |
|  |  | **JY293** | **M201** |  | **Means±SD** | **Range** |
| **TGW(g)** | 2009 | 23.26±0.52 | 62.71±0.23 | | 36.98±8.15 | 20.47-63.93 |
|  | 2012 | 24.56±0.15 | 69.2±0.15 |  | 36.86±7.74 | 23.38-59.62 |
|  | 2013 | 22.86±0.24 | 59.97±0.25 | | 33.25±7.67 | 15.83-58.69 |
|  |  |  |  |  |  |  |
| **GL(mm)** | 2009 | 8.11±0.31 | 13.60±0.18 | | 9.46±1.5 | 6.93-13.91 |
|  | 2012 | 7.94±0.20 | 14.32±0.37 | | 9.78±1.59 | 6.96-14.97 |
|  | 2013 | 7.80±0.27 | 13.58±0.28 | | 9.3±1.45 | 7.10-13.45 |
|  |  |  |  |  |  |  |
| **GW(mm)** | 2009 | 2.37±0.06 | 4.54±0.27 |  | 3.28±0.38 | 2.48-4.61 |
|  | 2012 | 2.68±0.12 | 4.19±0.16 |  | 3.41±0.42 | 2.30-4.93 |
|  | 2013 | 2.35±0.04 | 4.14±0.29 |  | 3.190.43 | 2.26-4.41 |
|  |  |  |  |  |  |  |
| **GLW** | 2009 | 2.30±0.08 | 3.42±0.04 |  | 2.93±0.55 | 1.79-5.36 |
|  | 2012 | 2.96±0.10 | 3.42±0.06 |  | 2.91±0.52 | 1.85-4.80 |
|  | 2013 | 2.45±0.13 | 3.38±0.02 |  | 2.96±0.56 | 1.91-4.85 |
