# Supplementary material for: Rapid Identification of Major QTLs Associated with Rice Grain Weight and Their Utilization
Source: PLoS One. 2015 Mar 27;10(3):e0122206. doi: 10.1371/journal.pone.0122206 (PMC4376791; doi:10.1371/journal.pone.0122206)
Supplement: S2 Table — (DOCX) [file pone.0122206.s004.docx]

**S2 Table**. No. of candidate genes in the hot regions

| **ID** | **Chromosome Starta** | **Enda** | **size** | **marker Num** | **Gene Num** |
| --- | --- | --- | --- | --- | --- |
| **1** | chromosome03 24,600,000 | 24,850,000 | 0.25M | 4 | 22 |
| **2** | chromosome03 25,000,000 | 25,350,000 | 0.35M | 11 | 36 |

a In order to avoid missing genes, both end of the region were measured to extra 1 to 5 Kb (kilo base pairs)
