# Supplementary material for: Rapid Identification of Major QTLs Associated with Rice Grain Weight and Their Utilization
Source: PLoS One. 2015 Mar 27;10(3):e0122206. doi: 10.1371/journal.pone.0122206 (PMC4376791; doi:10.1371/journal.pone.0122206)
Supplement: S3 Table — (DOCX) [file pone.0122206.s005.docx]

**S3 Table.** CAPS, dCAPs and InDel markers used for linkage mapping

| **Primer name** | **Primer sequence (5’-3’)** | **Type** | **Enzyme** | **Product size(bp)** |
| --- | --- | --- | --- | --- |
| SLAF13382F | CAGCAGGTTCTCCTCATGCC | dCAPS | *EcoR*II | 185,165/20 |
| SLAF13382R | TAGGCATTGCATCTCCCGTC |  |  |  |
| SLAF13411F | CACGCTCCAACCAAACATGA | dCAPS | *Mbo*II | 465,120/345 |
| SLAF13411R | TGAGTGGGACTCCGATAACGA |  |  |  |
| SLAF13430F | ACCATGCTTTTAATGTAATCTTCCT | dCAPS | *EcoR* II | 389, 24/365 |
| SLAF13430R | GCCTTGCAGATCATCCCGACACC |  |  |  |
| SLAF 13474F | GGCCACCGGCCACCATTACC | InDel |  | 506/451 |
| SLAF 13474R | AGCGGAGGCGGTGGAGCTTA |  |  |  |
| SLAF13482F | AATACCCATCAGAGATGTGACTTTG | dCAPS | *Tth111*I : | 132,21/111 |
| SLAF13482R | ATTGCTCGAGTCACTACGCC |  |  |  |
| GS3NF | CGAGGGAGATGGGCATGTGTT | InDel |  | 226/187 |
| GS3NR | GCCCAGCTTTTTTTTTCCTCCA |  |  | (*GS3/gs3*) |
| GLLF | GATTCTATCTGGTTCAGTGGTAGA | dCAPS | *Acc*I | 137,21/126 |
| GLLR | ACCGCCGTGTAAGTTCAACA |  |  | (*gl3/GL3*) |
