# Supplementary material for: Rapid Identification of Major QTLs Associated with Rice Grain Weight and Their Utilization
Source: PLoS One. 2015 Mar 27;10(3):e0122206. doi: 10.1371/journal.pone.0122206 (PMC4376791; doi:10.1371/journal.pone.0122206)
Supplement: S4 Table — (DOCX) [file pone.0122206.s006.docx]

**S4 Table**. SNPs detected in the coding sequence of *qGL3*

|  | **GL(mm)** | **Subpopulation** | **+1092** | **+1495** | **+2643** | **+2838** | **reference** |
| --- | --- | --- | --- | --- | --- | --- | --- |
| **M201** | 14.32 | *indica* | A | C | G | C | This study |
| **JY293** | 7.95 | *indica* | C | T | A | T | This study |
| **WY3** | NA(large) | *japonica* | A | T | G | C | Qi et al. 2012 |
| **FAZ1** | 9.22 | *indica* | C | C | A | T | Qi et al. 2012 |
| **CW23** | 14.64 | *japonica* | A | NA^a^ | NA | NA | Hu et al. 2012 |
| **PA64** | 9.05 | *indica* | C | NA | NA | NA | Hu et al. 2012 |
| **N411** | 14.15 | *japonica* | A | T | NA | NA | Zhang et al. 2012 |
| **N643** | 8.81 | *indica* | C | C | NA | NA | Zhang et al. 2012 |
| **9311** | 9.81 | *indica* | C | C | NA | NA | Zhang et al. 2012 |
| **Nipponbare** | 7.46 | *japonica* | C | T | NA | NA | Zhang et al. 2012 |

^a^ NA means not known
