# Supplementary material for: Rapid Identification of Major QTLs Associated with Rice Grain Weight and Their Utilization
Source: PLoS One. 2015 Mar 27;10(3):e0122206. doi: 10.1371/journal.pone.0122206 (PMC4376791; doi:10.1371/journal.pone.0122206)
Supplement: S5 Table — (DOCX) [file pone.0122206.s007.docx]

**S5 Table.** Correlation analysis among rice grain weight related traits (under the diagonal: 2009, upper the diagonal: 2012， the Coefficient of the same trait between the two years is in bold)

|  | **TGW** | **GL** | **GW** | **GLW** |
| --- | --- | --- | --- | --- |
| **TGW** | **0.910***** | 0.835*** | 0.366*** | 0.497*** |
| **GL** | 0.781*** | **0.928***** | 0.077 | 0.806*** |
| **GW** | 0.440*** | 0.230** | **0.608***** | -0.514*** |
| **GLW** | 0.441*** | 0.771*** | -0.415*** | **0.842***** |
