# Supplementary material for: Rapid Identification of Major QTLs Associated with Rice Grain Weight and Their Utilization
Source: PLoS One. 2015 Mar 27;10(3):e0122206. doi: 10.1371/journal.pone.0122206 (PMC4376791; doi:10.1371/journal.pone.0122206)
Supplement: S6 Table — (DOCX) [file pone.0122206.s008.docx]

**S6 Table.** ANOVA analysis of the major QTLs for grain yield related traits (2009)

| **Source** | **TGWa** | **GL** | **GW** | **GLW** |
| --- | --- | --- | --- | --- |
| ***GS3*** | 1059.64*** | 50.08*** | 0.01 | 5.02*** |
| ***qGL3*** | 1236.14*** | 70.90*** | 0.06 | 5.57*** |
| **RM571** | 1077.02*** | 35.32*** | 0.01 | 3.09*** |
| **Totalb** | 0.43 | 0.60 | 0.00 | 0.40 |

a *** indicates significance at P<0.001

bTotal means total variations explained by three QTLs
